# Temporal Integration Windows for Naturalistic Visual Sequences

**DOI:** 10.1371/journal.pone.0102248

**Published:** 2014-07-10

**Authors:** Scott L. Fairhall, Angela Albi, David Melcher

**Affiliations:** Center for Mind/Brain Sciences, University of Trento, Trento, Italy; CEA.DSV.I2BM.NeuroSpin, France

## Abstract

There is increasing evidence that the brain possesses mechanisms to integrate incoming sensory information as it unfolds over time-periods of 2–3 seconds. The ubiquity of this mechanism across modalities, tasks, perception and production has led to the proposal that it may underlie our experience of the subjective present. A critical test of this claim is that this phenomenon should be apparent in naturalistic visual experiences. We tested this using movie-clips as a surrogate for our day-to-day experience, temporally scrambling them to require (re-) integration within and beyond the hypothesized 2–3 second interval. Two independent experiments demonstrate a step-wise increase in the difficulty to follow stimuli at the hypothesized 2–3 second scrambling condition. Moreover, only this difference could not be accounted for by low-level visual properties. This provides the first evidence that this 2–3 second integration window extends to complex, naturalistic visual sequences more consistent with our experience of the subjective present.

## Introduction

The brain integrates incoming sensory information not only over space and sensory modality but also over time. Due to the diverse nature of this information, temporal integration mechanisms may vary across different timescales. The integration of environmental events into a unitary percept may occur within a few hundreds of milliseconds [1]. However, the integration of more complex information that unfolds over time may utilize neural mechanisms that operate across longer time scales. While varied mechanisms may operate over periods as long as minutes [2], [3], evidence has accumulated to suggest that temporal integration windows (TIWs) of 2–3 seconds may be a fundamental component of human cognition [4], [5].

One of the main tasks of the perceptual system is to parse the continuous input from the senses into meaningful objects and events. This goal necessarily involves integrating information over time. In the case of a real-world objects, for example, the influx of relevant information coming from the different senses at different sensory delays, such as vision, audition and touch, are combined over time in order to define the object as a unique spatiotemporal entity. Individuating and recognizing objects is thought to take on the order of 150 to 200 milliseconds [1], [6]. The fact that information is combined over time can be seen in judgments of simultaneity [7], [8] as well as in visual masking studies in which a target and mask are perceptually combined even though they are discrete events. A useful metaphor for describing these periods in which sensory input is combined is a “temporal integration window” [4], [5]. If two stimuli fall within the same temporal window then they are integrated into a coherent percept, while if the two stimuli fall in different windows then they are perceived as separate objects/events [1], [9].

Perception of complex phenomena such as motion [10], apparent motion [11], [12], biological motion [13] and events [2], [3], [14] requires temporal integration over longer periods of time because these entities are, by definition, extended in time. In the case of apparent motion, for example, if two brief stimuli are separated by less than a few hundred milliseconds, observers tend to perceive smooth and continuous motion in between the two discrete stimuli [11], [12]. In particular, evidence has accumulated to suggest that temporal integration windows (TIWs) of 2–3 seconds may be a fundamental component of human cognition [4], [5]. This 2–3 second integration window appears to be a ubiquitous feature of human cognition rather than being specific to particular cognitive processes or perceptual/motoric contexts. Both auditory and visual temporal intervals can be reproduced with high fidelity and with little across-trial variability up until 2–3 seconds, before the capacity to accurately represent these intervals breaks down [15], [16]. Likewise, the capacity to produce precise anticipatory motor actions synchronized with predictable auditory cues fails as inter-stimulus intervals exceed 3 seconds [17]. This phenomenon reflects more than a simple timing mechanism. The accumulation of evidence that allows the detection of motion coherence embedded within noise also asymptotes around 2–3 seconds as the integration mechanism reaches capacity [10], [18]. Moreover, the 2–3 second TIW extends not only to perception but also language and motor production. Speech utterances have been reported with a duration clustered around 2.5 seconds [19] and cross-cultural ethological studies have documented that the natural performance of motor actions is segmented within a 2–3 second window [20].

The prevalence of the 2–3 TIW across modalities, tasks, perception and production has led to the suggestion that it may reflect a general organizing principle of human cognition - better defined as the ‘subjective present’ i.e. the phenomenal impression of ‘nowness’ [5], [21]. This is an intriguing possibility but an important test of any such claim is whether this TIW can be observed in the processing of stimuli that more closely match our subjective experience. Normal perceptual experience involves a rich and tumultuous barrage of information while processes such as the reproduction of a tone or the accumulation of coherence information do not. In order to more closely approximate the complexity of real life, movie clips may act as a useful, if not perfect, proxy for our day-to-day subjective experience. In particular, movies typically contain multiple objects, motion-paths and events as well as shifts of attention (and gaze) as stimuli become more or less salient. If the function of the 2–3 second TIW is to integrate complex sequences of events into a coherent conscious stream then movie-clips present an important test of whether this integration window is an aspect of our subjective experience.

The goal of the present study is to address whether the 2–3 second temporal integration window extends to complex stimuli more consistent with our subjective experience. According to the logic of a temporal window, the brain should be able to combine information within the limits of a single TIW, even when the order of that information is scrambled, but have difficulty when information is scrambled over longer timescales. A similar approach has been used to identify differences between native and non-native speakers in integration capacity for phonetic sounds in spoken language [22]. We used movie clips and temporally shuffled the sequence of events over a range of different scales, from a few hundred milliseconds to several seconds. We hypothesize that if the 2–3 second TIW is indeed critical for understanding events in the subjective present, then there should be a dramatic increase in the subjective impression of the difficulty of following the movie as the duration of the window of temporal scrambling increases beyond the 2–3 second time period.

## Methods

### Participants

There were 15 participants in Experiment 1 (mean age: 23.4, 12 female) and 28 separate participants in Experiment 2 (age: 24.1, 18 female). The numbers were predetermined to allow complete counterbalancing of the videos in each condition. All gave written informed consent and received a small monetary compensation for participating. This study was approved by the Ethics Committee of the University of Trento in accordance with the provisions of the World Medical Association Declaration of Helsinki.

### Stimuli

On each trial, stimuli consisted of 12.8 second videos (90 in experiment 1, 98 in experiment 2). Video segments were selected from a pool of 7 relatively obscure international movies and the audio component was removed. First, 424 random clips were selected. One experimenter (A.A.) rated each video on a seven-point scale for the presence of a simple narrative. Of these, the best 100 were selected. Ninety were used in Experiment 1, 98 in Experiment 2).

Videos were presented at a frame rate of 25 Hz and a resolution of 360 by 272 pixels (subtending approximately 28° horizontally and 21° vertically). In order to better control for differences in low-level visual change across conditions, we introduced a visual transient every 5 frames. This manipulation was necessary because temporal scrambling in itself produces transients. By introducing the transient uniformly every 200 msec it is possible to balance the overall occurrence of transients across shorter and longer temporal scrambling intervals. Thus, across all conditions, videos were divided into *base-units* of 5 frames (i.e. 200 msec) where the 4^th^ frame was faded (RGB values halved) and the fifth frame was replaced by a blank frame.

Stimuli were shuffled within different time windows in order to see whether there was a discontinuity in perception when TIWs exceed around 2 seconds. To manipulate temporal integration demands, a segment of a fixed duration was taken and segments of the video were shuffled within this time window. For instance, for a 1600 msec TIW, sections of the video within the first 1600 msec were shuffled across time. Then the process was repeated across the next 1600 msec until the end of the video. Temporal shuffling was random with the exception that no segment followed its original predecessor.

To manipulate the overall level of shuffling independently of the TIW duration, we introduced the concept of a ‘shuffle-chunk’. Videos were shuffled *within* TIWs to different extents. For instance, in the 1600 msec TIW example, videos might be shuffled in either 200 or 400 msec chunks – thus the shuffling could be twice as frequent in the 200 msec condition (8 segments) than the 400 msec condition (4 segments) while preserving the overall duration of the TIW. This manipulation was important in order to vary the overall amount of shuffling and the *temporal* integration demands.

### Procedure

Experiments were run on a PC computer and controlled by Matlab (The MathWorks, Inc., Natick, Massachusetts, United States) using Psychtoolbox [23], [24]. Each video clip was presented only once to each participant (Experiment 1: 90 trials; Experiment 2: 98 trials) in one of the TIW/shuffle-chunk combinations. Videos were counterbalanced across subjects such that each video was seen an equal number of times at each level of the TIW/shuffle-chunk factorial design. The number of trials per participant within each cell of the factorial design was 6 in Experiment 1 and 7 in Experiment 2. We collected a subjective rating [25], [26], [27] of the subjects’ impression of how effortful the video was to watch. Specifically, participants rated the difficulty to follow (DtF) of each movie clip on a nine-point scale, where 1 indicated easy to follow and 9 indicated very difficult to follow.

To encourage vigilance, on 25% of trials the subjects also reported the basic narrative of the video using an open-ended keyboard response. These open-ended responses were not analyzed.

### Normalization of Ratings Across Participants and Videos

To standardize individual differences in usage of the rating scale, all responses were normalized such that each subject had a mean of 0 and a standard deviation of 1 across all responses made by that subject. In addition, to normalize differences in the innate difficulty to follow of each video segment, ratings of the same video across subjects were normalized such that each video had a mean rating of 0 and a standard deviation of 1.

### Exclusion of Potential Confounds

In order to allow for more naturalistic stimuli, we used movie clips rather than highly controlled but artificial stimuli. The videos were selected to be representative of the types of video clips we generally view and contained a wide range of cuts (between 0 and 11) with a mean shot-duration of 2.78 seconds and a broad distribution (standard deviation: 4.96 seconds). Inevitably, confounds may arise that interact with condition of interest. For example, visual change was likely to increase with the length of TIWs, as increasingly disparate sections of a video-segment are placed next to one another. We calculated the variation of potential confounds with our experimental manipulations for use in subsequent analyses.

To determine visual change, videos were down-sampled to a 5 vertical by 7 horizontal grid. This matrix was then vectorised and, for N-1 frames, each frame was correlated with its succeeding frame (skipping the blank frame occurring every 5^th^ frame). The result gave an acute measure of visual change (1-r). A log linear relationship was seen between TIW duration and degree of visual change (Exp 1: R^2^ = .21; Exp 2: R^2^ = .41). An approximately linear relationship was seen between cluster-chunk duration and visual change (Exp 1: R^2^ = –.56; Exp 2: R^2^ = –.60).

The interaction between experimenter cuts and ‘natural’ cuts (termed *cross-cuts*) in the video was determined using the visual change process above. An inter-frame threshold of r<.6 was capable of detecting cuts of both a natural and experimental origin while being insensitive to camera pans. This process revealed that a log linear relationship was again present between TIW and cross-cuts (Exp 1: R^2^ = .35; Exp 2: R^2^ = .65). A linear effect was seen between shuffle-chunk and cross-cuts ((Exp 1: R^2^ = .36; Exp 2: R^2^ = .17). Changes in mean luminance were not correlated with either independent variable (p-values>.2).

Effects of visual change and cross-cuts were controlled using mean adjustment [28]. First, the covariance at the group level between visual change and DtF at each level of the factorial design was determined. Mean visual change for each condition (e.g. TIW: 1600, shuffle-chunk: 400 msec) was determined across video. Covariance was accounted for by regressing each condition against the mean DtF for that condition averaged over participants. The variation accounted for by this potential confound was removed from the data [i.e. DtF_adj_ = DtF-*b*(VC_ij_–mean(VC)); where *b* is the regression coefficient between DtF and visual change]. This process was repeated on the adjusted data now using the variable cross-cuts (accounting for potential colinearity between visual change and cross-cuts covariates).

### Data Availability

Individual subject and video data are available as supporting information (File S1).

## Results

### Experiment 1

**Figure 1:**
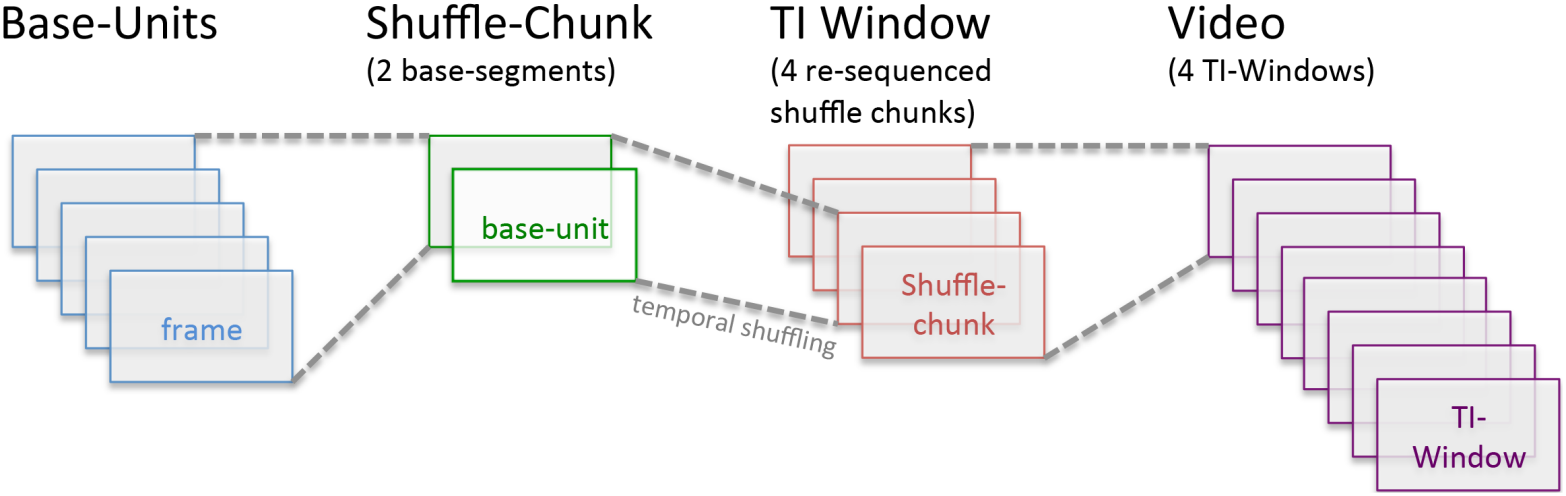
Example of a video re-sequencing for a 1600 msec TIW, 400 msec shuffle-chunk condition. Across all conditions, the base-unit was 5 frames (200 msec). For 400 msec shuffle chunk conditions, two sequential base-units would be combined. In this example, a 1600 msec TIW would thus consist of 4 re-ordered shuffle-chunks. Finally, for 1600 msec TIWs, this process would be repeated 8 times to produce the entire 12.8 second video.

**Figure 2:**
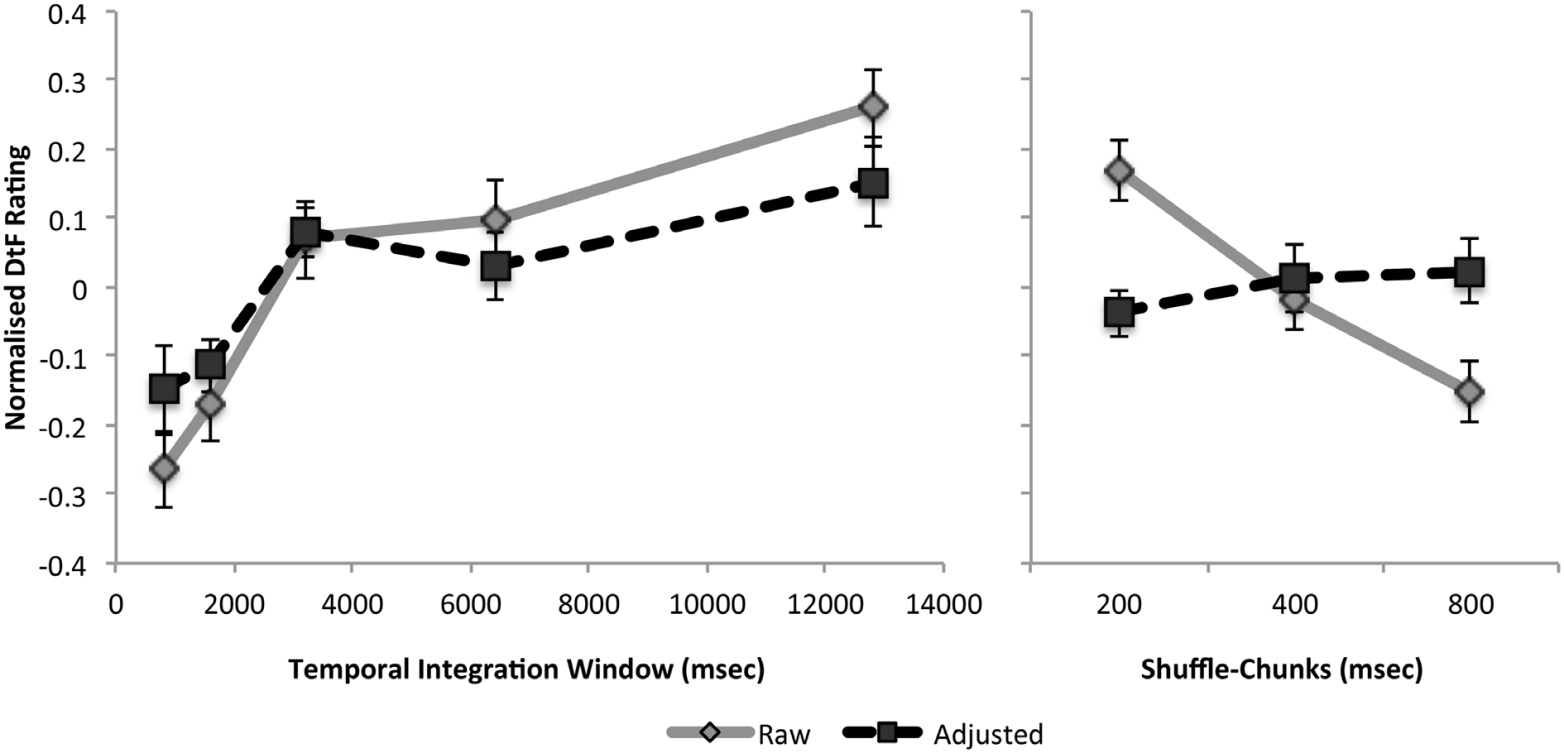
Difficulty to Follow (DtF) as a function of Temporal Integration Window (left) and Shuffle-Chunk (right). Data are presented both before and after mean-adjustment for low-level visual features (visual change and cross-cuts). Note the pronounced increase in difficulty to follow rating between 1600 and 3200 msec that persists after adjustment for the low-level visual properties of the movie clips. Also note that the effect of shuffle-chunk (the degree of temporal shuffling within a TIW) can be alternatively accounted for by low-level visual features.

As hypothesized, the difficulty to follow (DtF) ratings were influenced by the time window of shuffling. A two-way repeated measures ANOVA revealed a significant main effect of the factors TIW (F_(4,56)_ = 14.5, 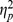 = .23, p<.001) and shuffle-chunk (F_(2,28)_ = 14.0, 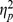 = .12, p<.001) but no interaction between them (F<1). The pronounced increase between 1600 and 3200 msec in DtF can be seen in Figure 1 (T_(14)_ = 4.2, p<.001, Cohen’s d = 1.07, one-tailed). In terms of our 9-point scale, effects were modest. The grand averages for the five TIWs depicted in figure 2 extended over a ∼1.5 point range of the original 9-point scale. However, in terms of relative changes across the TIW range, the increase in DtF between 1600 and 3200 was pronounced - approximately one and a half times (146%) the next greatest increase (that occurring between 6400 and 12800 msec; see figure 2). This effect became more evident once DtF ratings were mean-adjusted using the covariates visual change and cross-cuts. Following adjustment, only the hypothesized difference between 1600 and 3200 msec remained significant (T_(14)_ = 3.37, Cohen’s d = 0.87, p = .004, one-tailed–see dotted line).

The effect of shuffle-chunk within TIWs is presented in the right panel of Figure 1. It is interesting to note that the approximately linear trend between shuffle-chunks and DtF was fully accounted for by visual change (dotted line). Thus, in contrast to the stepwise increase in DtF between 1600–3200, both the degree of shuffling within windows (shuffle-chunks) and changes in DtF at longer or shorter intervals could be alternatively explained by low-level visual properties.

The first data point in figure 2 contains an unscrambled video condition (800 msec TIW and 800 msec shuffle-chunk). It is plausible that the unscrambled video acts as an outlier distorting the data. However, this appears not to be the case. At the 800 msec shuffle-chunk, the difference between unscrambled and the next highest TIW (1600 msec) is significant (t_(14)_ = 1.96, Cohen’s d = 0.53, p = .036, one tailed) but is less than the difference over the critical 1600 to 3200 msec TIW increase (t_(14)_ = 2.11, Cohen’s d = 0.60, p = .027, one tailed).

### Experiment 2

To provide an internal replication and to refine our estimate of the TIW, a follow up study was conducted using TIWs of 1200 2000 2800 3600 and 4400 msec and 200 and 400 msec shuffle-chunks. Additionally, a number of trials with unscrambled and fully scrambled (12800 msec) TIWs were included in order to peg responses over a similar range to Experiment 1 but were not included in the analysis.

**Figure 3:**
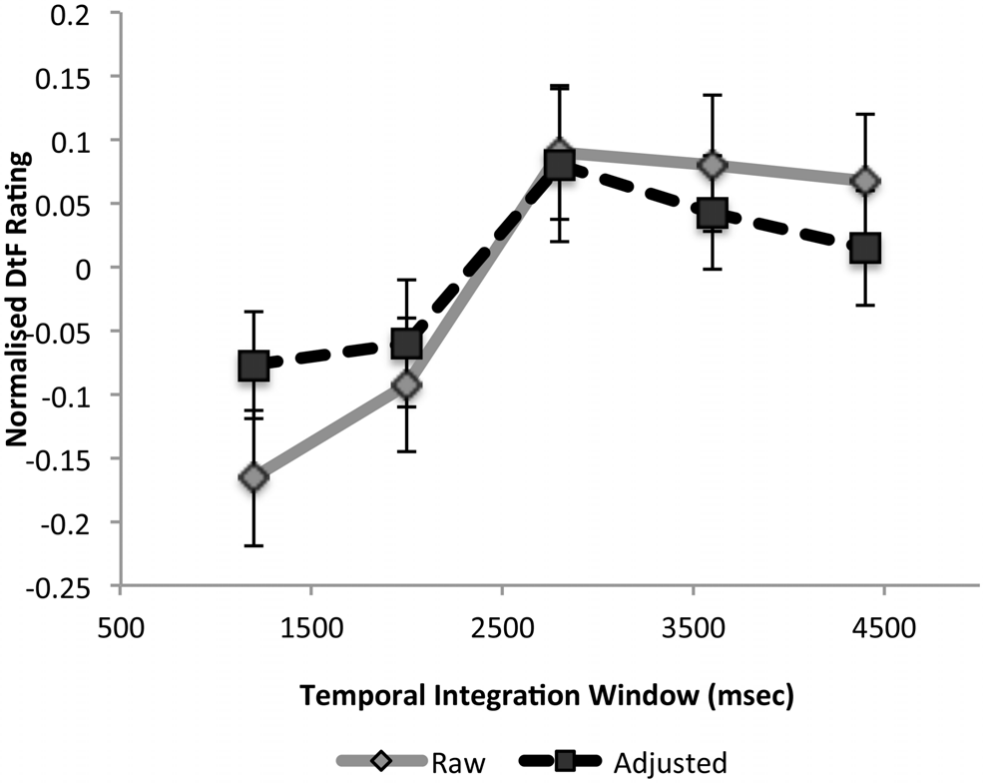
Difficulty to Follow (DtF) as a function of Temporal Integration Window for Experiment 2. Data are presented both before and after mean-adjustment for low-level visual features. As in Experiment 1, the sharp increase in DtF between 2000 and 2800 msec persists after mean-adjustment for low-level visual features of the video clips.

Results (see figure 3) were consistent with Experiment 1. Both TIW (F_(4,108)_ = 4.9, 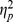 = .07, p<.001) and short-shuffle F_(1,27)_ = 20.9, 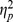 = .08, p<.001) had a significant influence on DtF and there was no interaction between these factors (F<1). The hypothesized stepwise increase in DtF was again apparent between 2000 and 2800 msec (T_(27)_ = 2.4, Cohen’s d = 0.49, p<.01, one-tailed). This increase was more than twice (220%) the next greatest increase, which occurred between 1200 and 2000 msec TIWs. This increase remained significant after mean-adjustment for the covariates visual change and shuffle-chunk (t_(27)_ = 1.99, Cohen’s d = 0.38, p = .028, one-tailed). The influence of shuffle-chunk did not survive adjustment for visual change.

### Item analysis

The preceding statistical analyses indicate generalizability to the population but would our effects extend to different sets of videos? To address this we reran the main analysis now considering video clips rather than participants as the random factor. The main effect of TI-window was significant (Exp 1: F_(4,356)_ = 14.4, 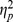 = .14, p<.001; Exp 2: F_(4,388)_ = 8.3, 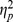 = .08, p<.001), Furthermore, as in the preceding analyses the only significant increases in DtF were between 1600 and 3200 msec (Exp 1: t_(89)_ = 2.8, Cohen’s d = 0.29, p<.005) and between 2000 and 2800 msec (Exp 2: t_(97)_ = 3.2, Cohen’s d = 0.25, p<.001). This indicates generalizability to videos sampled in the same manner although it is an open question whether 2–3 second TIW effect would extend to different forms of video (e.g. highly familiar clips, Hollywood trailers).

We additionally considered whether the DtF of the unscrambled version of the video influenced the 2–3 second TIW effect. To do this, we determined the fit of each video to a function modeling a single stepwise increase at the hypothesized TIW. Then we determined the correlation between model-fit and the DtF rating of the unscrambled video. In neither experiment did the TIW effect vary as a function of the DtF of the original clip (Exp 1: R^2^ = .019; Exp 2: R^2^ = .034; p-values>.05).

## Discussion

In the present study we investigated whether a 2–3 second TIW operated over the processing of naturalistic visual sequences. In two experiments, we presented 12.8 second video clips manipulated to vary temporal integration demands within and across this proposed window boundary. In both studies, rather that a simple linear increase in the subjective difficulty to follow with temporal scrambling, a dramatic increase in DtF was evident between 2 and 3 seconds. This provides evidence that there is a natural integration window of around 2 seconds that operates even in a very different context to those previously studied, extending to complex multidimensional visual streams more consistent with the subjective experience.

The pattern of responses in both experiments was consistent with a temporal integration mechanism that easily re-integrates sequences within 2 seconds, is progressively strained as the integration window is extended from 2000 to 2800 msec, and then reaches capacity after 2800 msec. This pattern of responses is in accord with previous studies of unidimensional temporal integration. For example, the anticipatory response to predictable auditory cues is relatively flawless until inter-stimulus intervals of about 1200 msec, then progressively breaks down from 1800–3600 msec, after which the anticipatory mechanism fails [17]. Similarly, the capacity to integrate two briefly presented motion coherence fields shows an abrupt reduction in efficiency when inter-stimulus intervals lengthen beyond around 2 seconds [18]. The consistency between these results and the pattern of responses seen in the present study suggests that a similar mechanism (or a similar constraint) underlies the temporal integration of simple unidimensional and complex multidimensional stimuli.

Of note, in neither study was there an interaction between the amount of within-window shuffling (shuffle-chunks) and the effect of TIW duration. For instance, in Experiment 1 there is a linear increase in DtF with within-window shuffling that is independent of the TIW duration. A similar monotonic increase is observed in the across-subject variability of eye movements with scrambled videos [29]. This indicates that two separate mechanisms account for the effect of temporal shuffling on DtF ratings. The first may reflect the overall alteration in the incoming visual sequence and correlate with oculomotor variability while the second independent mechanism instead appears to reflect the time period over which the information must be re-integrated. Thus the 2–3 second integration effect can be seen to be independent of the overall amount of temporal scrambling.

Critically, the 2–3 second temporal integration effect could not be explained by low-level aspects of the videos (visual change and cross-cuts). It is not possible to determine with complete certainty whether the true pattern of results reflects the adjusted (dotted lines in figures 2 & 3) or unadjusted data (gray lines in figures 2 & 3). Low-level visual properties may be only incidentally correlated with our temporal shuffling procedure and irrelevant to the integration process. On the other hand, there may be two complimentary processes at play – one captured by visual change and the other by temporal integration processes. The influence of the short-shuffle chunks discussed in the previous paragraph (also accountable by low-level video properties) suggests the latter case might be true. Specifically, our results suggest that the effect of low-level change on DtF operates independently of the temporal integration process. In either case, both the adjusted and unadjusted data indicate that different processes are occurring before and after the 3-second integration period.

The current findings provide further evidence for a key integration window of around 2–3 seconds [5]. However, studies of working memory span [30], as well as neuroimaging studies of the integration of narrative elements over different time scales [2], [3] suggest that longer time scales may also play an important role in understanding the plot of films. We think it is likely that such long time scales would involve different mechanisms and neural substrates from the 2 to 3 second TIW studied here.

It is interesting to note that although the duration of individual shots in Hollywood films varies greatly, it is rare to find shots less than around 2 seconds [31] (see www.cinemetrics.lv). Movie trailers and action sequences tend to have relatively short shot durations (a larger number of cuts per minute) of around 2–3 seconds (the average shot length in a Michael Bay film is 3.0 seconds: www.cinemetrics.lv), while tracking shots of several minutes can also be found, for example, in the work of director Alfonso Cuarón. Given that people typically move their eyes several times per second, even the shortest shots are usually an order of magnitude longer than human fixation durations while reading. Nonetheless, movie shots have often been compared to fixations by film theorists and directors [32]. This raises the question of why film is so “inefficient” compared to a human fixation and why there are not several cuts per second in typical movies. One possibility, consistent with the current results, is that event information is accumulated over a period of a few seconds, making clip durations of 2–3 seconds an ideal compromise between efficiency (showing as many different shots as possible in a short period of time) and ease of viewing.

In order to perceive coherent objects and events, the brain integrates incoming sensory information over time, over space and across sensory modalities. It is known that temporal integration mechanisms operate across multiple timescales [1]. Integration windows of around 100–150 ms are found in various paradigms, including backward masking and motion integration [4]. Similarly, a number of other studies have reported integration windows of around 300 ms for phenomena such as apparent motion, the attentional blink and inhibition of return [33]–[35]. However, one of the most apparent, yet mysterious features of the stream of consciousness is that there is an integrated subjective present, which has been estimated to extend for around 2 to 3 seconds [5], [21]. Most studies of conscious awareness have focused on much shorter time windows involved in tasks such as detection of a single stimulus. In contrast, the subjective present seems to involve an aspect of consciousness that is extended in time. Here, we examined the role of this time window in our understanding of a complex, multidimensional stimulus that is more consistent with our subjective experiences of the world. Overall, these results suggest that a function of this 2–3 second window may be to provide a stable and coherent representation of events in a complex, ever-changing world.

## Supporting Information

File S1 **Individual subject and video data for experiments 1 and 2.** This excel file includes mean DtF ratings for each subject and mean values for visual change and cross-cuts for each condition.
(XLSX)
